# Mr. Higgins’ Case of Traumatic Tetanus

**Published:** 1842-08-27

**Authors:** 


*Traumatic Tetanus: Cure.* By Mr. Higgins.—This was a decided case. The patient had wounded a finger with a straw-cutting instrument, on the 5th of February. On the night of the 21st, he slept in an exposed situation; and on the 25th, the author found him complaining of violent spasmodic pain at the epigastrium, with great difficulty of breathing; stiffness in the muscles of the neck, and inability to open the mouth; the pulse full, 120. He was bled to ℥xx, and on the succeeding day to ℥xxx, which relieved, but only temporarily. He had two grains of opium every three hours. On Sunday, he was in a state of complete opisthotonos, and intoxicated from the effect of the opium; but suffering little or no pain, when undisturbed; he could breathe, talk, and swallow easily. On Monday, his circumstances were exactly the same. Mr. H. found him in precisely the same position as he had left him in on the preceding day—that is, his body resting on his heels and head. He was again bled to ℥xvj. On the night of March 2, he had profuse perspiration, with great relief. The opisthotonos was so far gone as to allow the patient to turn on his side. At the date of report he was nearly well.—*Brit. and For. Med. Rev.* July, 1842. *From Lancet*, No. v. April 30, 1842.

[This case is extremely creditable to Mr. Higgins. Another case is reported in the Provincial Medical and Surgical Journal for April 30, 1842, No. iv., which was successfully treated by sesqui-carbonate of iron. In this case there was complete emprosthotonos, with incomplete trismus.]—*Rev.*

